# Acquired Bronchobiliary Fistula in a Young Adult Patient With Sepsis: A Case Report

**DOI:** 10.7759/cureus.53110

**Published:** 2024-01-28

**Authors:** Pallavi Pokharel, Sahar Panah, Robert J Dabek, Klara Schwarzova, Fawaz Araim, Alok Gupta

**Affiliations:** 1 Surgery, Ascension Saint Agnes Hospital, Baltimore, USA; 2 General Surgery, Ross University School of Medicine, Bridgetown, BRB; 3 General Surgery, Ascension Saint Agnes Hospital, Baltimore, USA

**Keywords:** endoscopic retrograde cholangiopancreatography (ercp), bbf, laporoscopic cholecystectomy, percutaneous cholecystostomy tube, bronchobiliary fistula

## Abstract

Bronchobiliary fistula (BBF) is a rare, highly morbid condition that results from an abnormal connection between biliary channels and the bronchial tree. In the past, this condition has been known to be caused by untreated hydatid cysts or hepatic abscesses that can erode through the diaphragm into the pleural cavity and bronchial tree, creating fistulation. However, the condition’s spectrum has changed in recent years, and BBFs have also become associated with neoplasm, iatrogenic causes, and trauma. Cases of BBF are treated differently, either with simple conservative management or invasive surgery. We present a case of a 46-year-old male initially presenting with sepsis, who was found to have a BBF. The diagnosis was made after a hepatobiliary iminodiacetic acid scan showed the flow of a tracer in the lung fields. The condition was likely due to acute cholecystitis and prior biliary instrumentation. The patient was treated successfully with percutaneous cholecystostomy tube insertion followed by elective laparoscopic cholecystectomy several weeks after hospital discharge.

## Introduction

Bronchobiliary fistula (BBF) is defined as an abnormal communication between the biliary system and bronchial tree, requiring well-planned management to decrease morbidity and mortality. Most patients present with infectious pneumonia, biliary obstruction, and expectoration of bile as a pathognomonic finding that can lead to acute pneumonitis and extensive lung injury [1,2]. The diagnosis is made based on presenting symptoms, clinical history, and imaging, and without proper management, it can result in serious complications and even death. Several mechanisms are suggested, such as local pulmonary infection, biliary lithiasis, cholecystitis, pancreatitis, liver/biliary tumors, and, rarely, hepatic trauma [3]. BBF has a high mortality and morbidity rate of 12.2%, making early detection and management imperative. Clinical diagnosis of BBF relies mainly on imaging and clinical features. The diagnosis can also be made by hepatobiliary iminodiacetic acid scan (HIDA) scan, endoscopic retrograde cholangiopancreatography (ERCP), magnetic resonance cholangiopancreatography (MRCP), or computed tomography (CT) scan [3]. HIDA scan and MRCP have the greater advantage of providing a definite diagnosis in patients who have no pathology of the biliary tract. Detection of bilirubin in the sputum can also help establish a diagnosis. Although there is no widely accepted guideline for the management of BBFs, therapeutic options include non-invasive biliary stent placement through ERCP, percutaneous drainage, or surgery as a definitive choice. ERCP or percutaneous drainage has been the most preferred choice for BBFs conventionally, and ERCP can be both diagnostic and therapeutic. Surgical interventions are also considered when nonsurgical therapies fail [4]. Based on published studies [1], ERCP or percutaneous drainage remains the priority in treating BBFs as they are a much safer and easier approach in this field. ERCP helps demonstrate the fistula tract and identify distal obstruction, which provides an advantage in patients requiring a stent placement or dilatation of the biliary tracts in cases of obstruction. Early diagnosis and management can alleviate a patient’s symptoms and improve outcomes. However, the effect of early detection and treatment on the overall prognosis and outcome of the disease remains unknown. Here we present a case of a patient who presented to a community hospital with pneumonia, sepsis, and acute cholecystitis and was diagnosed with BBF, which was successfully treated with endoscopic stenting and percutaneous drainage.

## Case presentation

**Figure 1:**
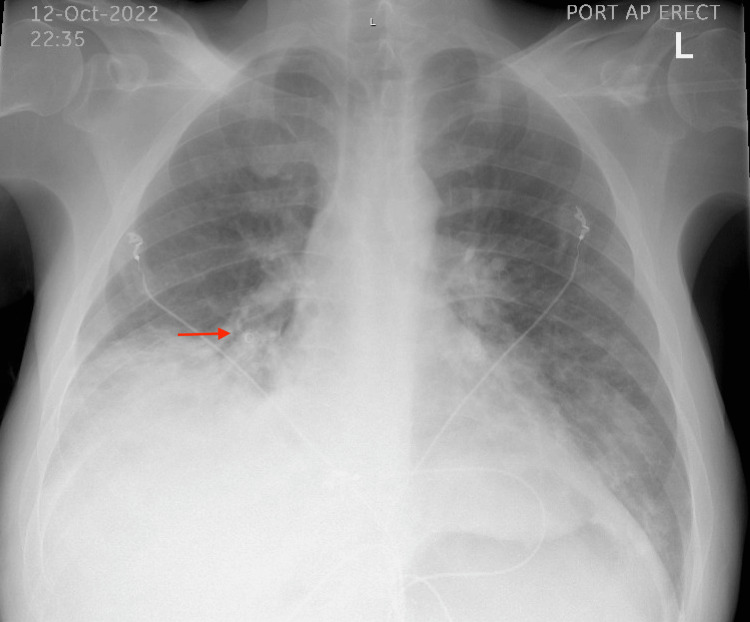
Anteroposterior chest X-ray showing right-sided multifocal pneumonia

A 46-year-old male with a past medical history of alcohol abuse, extensive history of gallstone pancreatitis complicated by pseudocyst formation, common bile duct (CBD) obstruction status postgastrocystostomy, multiple ERCP procedures with CBD stenting, type 2 diabetes, and bipolar disorder presented to the Emergency Department (ED) with chief complaints of productive cough, dyspnea on exertion and at rest, nausea, and vomiting for three days. Chest X-ray revealed right-sided multifocal pneumonia (Figure 1).

**Figure 2:**
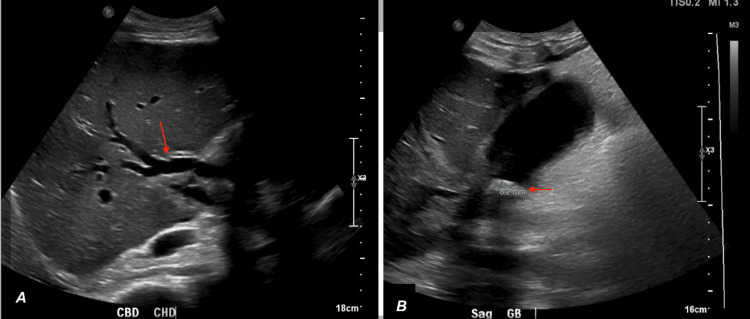
Abdominal ultrasound showing (A) intrahepatic biliary dilation and (B) gallstone at the neck of the gall bladder

In the ED, the patient was saturating 86% SpO_2_ on room air. Later, he was admitted to the intensive critical care unit (ICU) due to refractory hypoxia and acute respiratory distress syndrome, requiring intubation and mechanical ventilation. During the ICU course, bronchoscopy revealed right and left main bronchi coated with copious amounts of bilious appearing fluid, and abdominal ultrasound showed intrahepatic biliary dilation and gallstone at the neck of the gallbladder (Figure 2).

**Figure 3:**
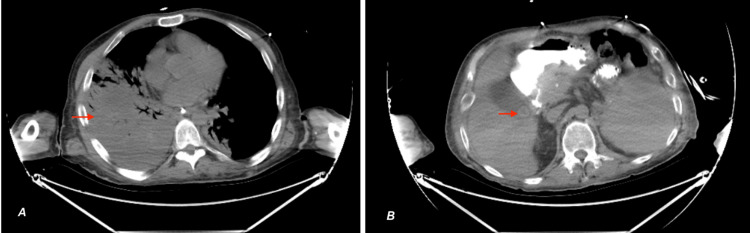
Axial CT image of the abdomen and pelvis without contrast demonstrating (A) multilobar consolidations and (B) stone in the gallbladder neck CT, computed tomography

Surgery was consulted due to concerns for a tracheoesophageal (TE) fistula, given the finding of bile on bronchoscopy. A contrast-enhanced CT of the chest, abdomen, and pelvis showed multilobar consolidations, cholelithiasis with gall bladder wall thickening, a 1.5-cm cholelith in the gallbladder neck, and no fistulous tract between the trachea and esophagus (Figure 3).

**Figure 4:**
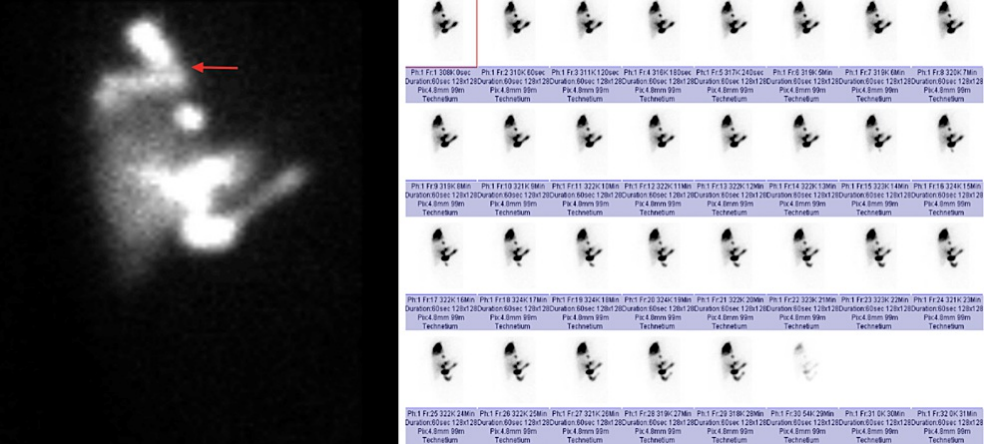
HIDA scan showing activity in the right chest indicating BBF BBF, bronchobiliary fistula; HIDA, hepatobiliary iminodiacetic acid scan

On hospitalization day (HD) 3, a HIDA scan (Figure 4) was performed, which showed activity identified in the right chest, compatible with a broncho biliary fistula, and no activity within the gallbladder, suggesting cystic duct obstruction.

**Figure 5:**
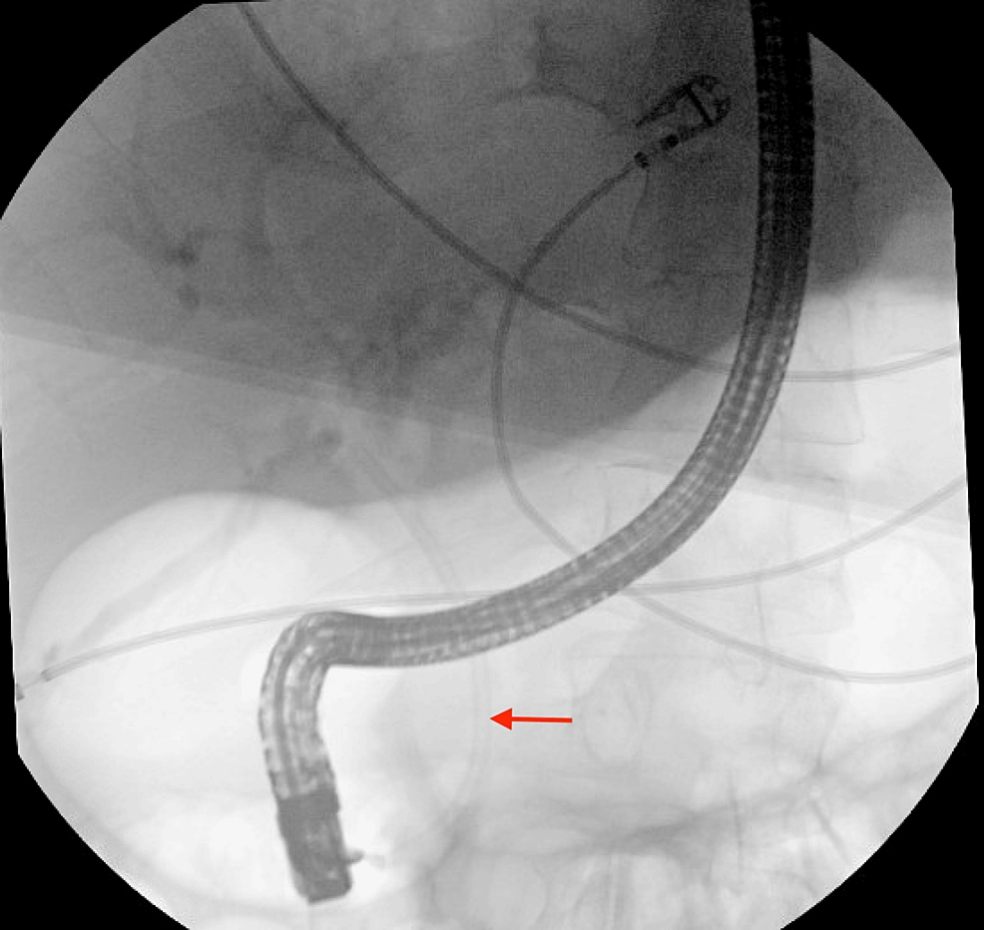
A plastic stent placement for bronchobiliary fistula

ERCP was performed, which showed a 15-mm stone in the CBD, which was extracted using a balloon. A plastic stent was then deployed for the management of the BBF (Figure 5).

**Figure 6:**
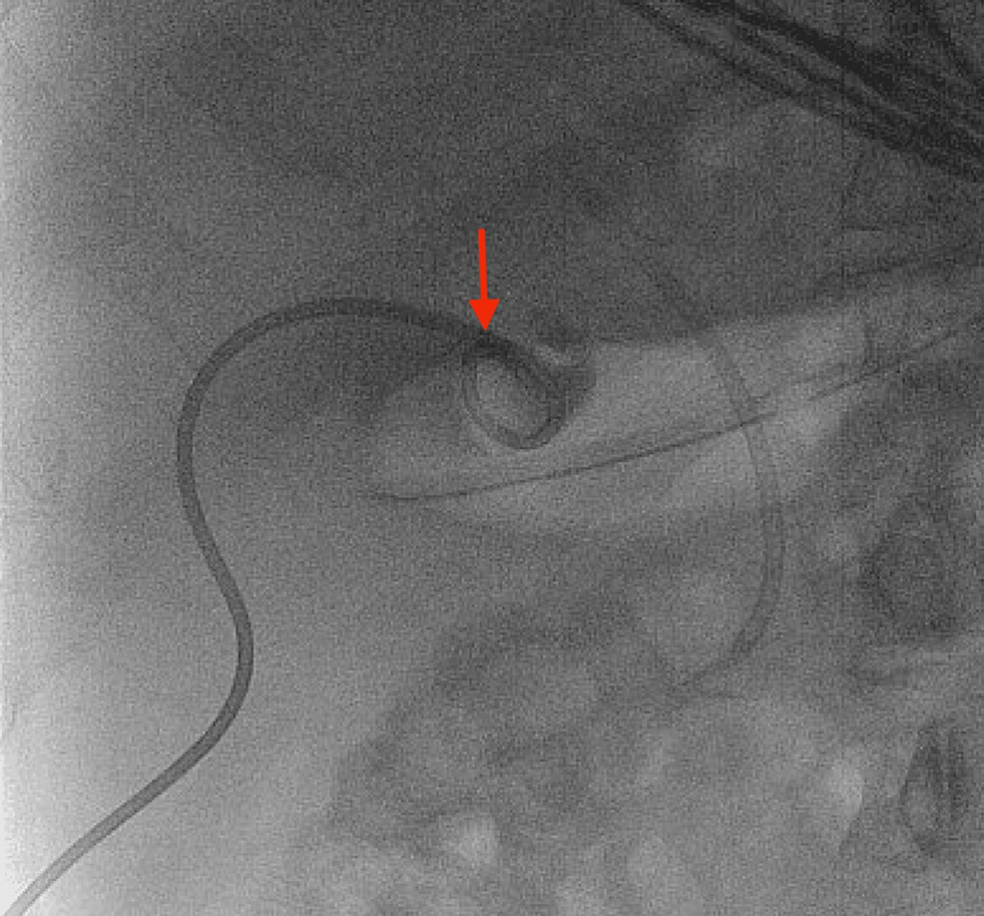
Percutaneous cholecystostomy tube placement

During the ICU course, the patient was successfully extubated. On HD 6, the patient underwent successful percutaneous cholecystostomy tube (PCT) placement (Figure 6).

**Figure 7:**
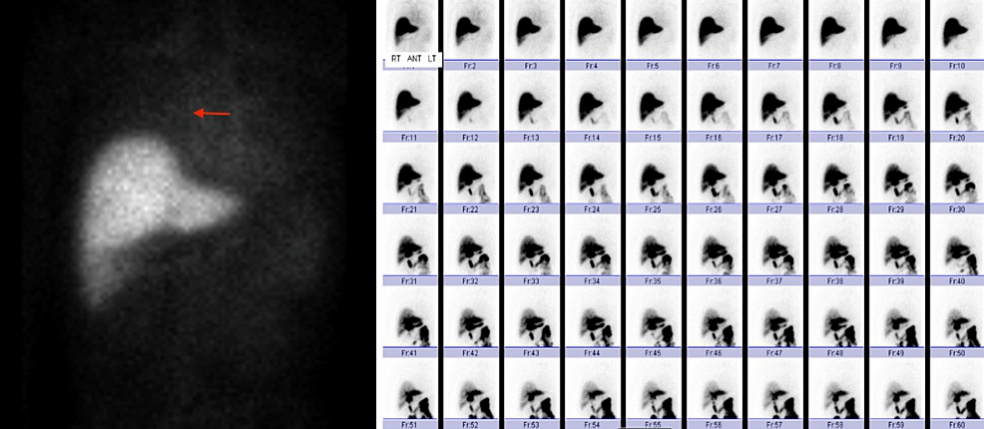
HIDA scan showing closure of the BBF BBF, bronchobiliary fistula; HIDA, hepatobiliary iminodiacetic acid

Afterward, the patient remained hemodynamically stable and was discharged on HD 10. At his four-week follow-up at the outpatient clinic, a repeat HIDA scan demonstrated closure of the BBF (Figure 7).

After two months, the patient successfully underwent laparoscopic cholecystectomy, extensive lysis of adhesions, and removal of the percutaneous cholecystostomy drainage tube.

## Discussion

BBF has two forms: congenital and acquired. Congenital BBF presents early in life with poor feeding, respiratory distress, and bilious sputum that is usually accompanied by other biliary tract abnormalities, whereas acquired BBF develops as a result of injury to the bronchioles or biliary tracts [5]. Hydatid cysts have been known as the main etiology in the development of BBFs. Due to early detection and broad-spectrum antibiotics availability, the incidence of BBF has been drastically reduced. Often, BBF can be easily misdiagnosed as infectious pneumonia, making its diagnosis difficult. Clinical manifestations of the condition include fever, productive cough, bile-colored sputum, chest or abdominal pain, and jaundice [5].

The current standard of care involves either nonsurgical or surgical management depending on the severity, size, and location of the fistula [6]. HIDA scan has been shown to be an effective non-invasive tool to detect BBF. In addition, pleural fluid and sputum analysis can also be used to make the diagnosis but requires a high index of suspicion. An ERCP is more practical for diagnosis than an MRCP since it can provide a precise diagnosis and treatment, with the goal of biliary decompression and stent implantation to ease bilious drainage. Even though there is still no optimal management for BBF, ERCP and percutaneous drainage are the most reliable nonsurgical methods for treatment [7]. Although ERCP has been shown to be very effective in managing BBFs, studies show that the failure rate of noninvasive management in BBFs without surgery is nearly 38%[6]. Therefore, in case of complications or failure of nonsurgical techniques, surgery can provide the desired outcome and resolution of BBF.

## Conclusions

When treating patients with BBF, nonsurgical interventions such as ERCP and PCT placement or bronchoscopy-guided embolization should be considered first, as they are both safe and effective. However, the outcome of these modalities depends on the status of the inflammatory process and the overall status of the patient. Surgical procedure is considered when interventional techniques have failed or if BBF has developed secondary to trauma or tumor. However, no surgical intervention was required in our case, ERCP and PCT procedures were used for the primary management of BBF, and the patient was treated successfully with a good outcome.
